# Maternal Biomarkers of Endothelial Dysfunction and Preterm Delivery

**DOI:** 10.1371/journal.pone.0085716

**Published:** 2014-01-22

**Authors:** Xinhua Chen, Theresa O. Scholl

**Affiliations:** Department of Obstetrics and Gynecology, Rowan University – School of Osteopathic Medicine, Stratford, New Jersey, United States of America; University of Catanzaro Magna Graecia, Italy

## Abstract

**Background:**

Endothelial dysfunction is key to the development of atherosclerosis. Preterm delivery foreshadows later maternal cardiovascular disease (CVD), but it is not known if endothelial dysfunction also occurs. We prospectively measured circulating biomarkers of endothelial dysfunction in pregnant women with preterm or term delivery.

**Methods:**

We conducted a case-control study nested within a large prospective epidemiological study of young, generally healthy pregnant women. Women who delivered preterm (<37 completed weeks gestation, n = 240) and controls who delivered at term (n = 439) were included. Pregnancies complicated by preeclampsia were analyzed separately. Circulating endothelial dysfunction biomarkers included soluble intercellular adhesion molecule-1 (sICAM-1), vascular cell adhesion molecule-1 (sVCAM-1) and soluble E-selectin (sE-selectin).

**Results:**

Elevated levels of sICAM-1 and sVCAM-1 were positively associated with preterm delivery independent of usual risk factors. At entry (∼16 wks), the adjusted odds ratio (AOR) was 1.73 (95% confidence interval (CI) 1.09–2.74) for the highest quartile of sICAM-1 versus the lowest quartile and for sVCAM-1 the AOR was 2.17 (95% CI 1.36–3.46). When analysis was limited to cases with a spontaneous preterm delivery, the results were unchanged. Similar results were obtained for the 3rd trimester (∼30 wks). Elevated sE-selectin was increased only in preterm delivery complicated by preeclampsia; risk was increased at entry (AOR 2.32, 95% CI 1.22–4.40) and in the 3rd trimester (AOR 3.37, 95% CI 1.78–6.39).

**Conclusions:**

Impaired endothelial function as indicated by increased levels of soluble molecules commonly secreted by endothelial cells is a pathogenic precursor to CVD that is also present in women with preterm delivery. Our findings underscore the need for follow-up studies to determine if improving endothelial function prevents later CVD risk in women.

## Introduction

Pregnancy may be a prognostic state unique to women that allows early identification and remediation of risk factors and ultimately the prevention of chronic diseases of later life [1], [2]. Serious complications (e.g. gestational diabetes) that signal a positive ‘stress test’ may also be markers for increased risk (e.g. type 2 diabetes) later on [3]. Cardiovascular disease (CVD) is a leading cause of death for women in the United States and CVD mortality is higher in US women than men [4]. Pregnancy complications, particularly preeclampsia, may presage susceptibility to later CVD [5], [6]. Preterm delivery, defined as birth occurring prior to 37 completed weeks' gestation, is more common than preeclampsia (2–8%) complicating 12–13% of all births in the United States [7]–[9]. Previous epidemiologic data suggest that a preterm delivery may foreshadow later maternal CVD [10]–[12].

Vascular endothelial cells are activated under conditions that occur during pregnancy (inflammation and oxidative stress) and increase expression of adhesion molecules and selectins [13]. These include the soluble intercellular adhesion molecule-1 (sICAM-1), the vascular cell adhesion molecule-1 (sVCAM-1) and soluble E-selectin (sE-selectin) [13], [14]. Endothelial dysfunction denotes an imbalance in vascular function. It is considered a key element in the development of atherosclerosis and associated with diabetes, insulin resistance, obesity and preeclampsia [14]–[17]. While invasive and costly non-invasive methods that directly measure endothelial function are available, circulating soluble forms of adhesion molecules and selectins produced by endothelial cells are widely used as biomarkers of endothelial dysfunction [14]–[17].

We hypothesized that endothelial dysfunction is an underlying pathological state that will manifest in women who deliver preterm and increase their risk of future CVD. Currently, it is unclear if these biomarkers are present in pregnant women who deliver preterm [18]. Thus we examined the association of circulating sICAM-1, sVCAM-1 and sE-selectin during early and late pregnancy with preterm delivery. Since preeclampsia is a serious complication of pregnancy and women with preeclampsia are at an increased risk for preterm delivery, we also examined associations in preterm delivery with and without preeclampsia.

## Materials and Methods

### Study Design

We conducted a case-control study nested within the Camden Study, a large prospective epidemiological study of young, generally healthy pregnant women residing in one of the poorest cities in the continental United States [19]. The cohort of study participants enrolled between 1996 and 2006 was recruited from among patients enrolling at the Osborn Family Health Center, Our Lady of Lourdes Medical Center and St John the Baptist prenatal clinic in Camden, NJ. The institutional review board at the University of Medicine and Dentistry of New Jersey approved the study protocol. Informed written consent was obtained from each participant at enrollment (on average at gestational week 13.56±4.87, mean ± SD) after explanation of the nature and purpose of the study. A total of 3.5% of the women who had serious non-obstetric problems (e.g. Lupus, type 1 or 2 diabetes, seizure disorders, malignancies, acute or chronic liver disease, drug or alcohol abuse and psychiatric problems) were not eligible. Eighty percent of the patients who were eligible agreed to participate. A total of 8.3% of participants dropped out after enrollment due either to a move from the area or to an early pregnancy loss. A final total of 2,379 participants whose pregnancy culminated in a live birth were used to select cases and controls.

Data on socioeconomic, demographic and lifestyle were obtained by interview at entry to care (14.2±4.01 gestational weeks, mean ± SD), and updated at gestational weeks 20 and 28. Participants were scheduled to see study research assistants before or after their regular prenatal visits at entry, gestational weeks 20 and 28. Interview data, dietary data and anthropometrics were collected in person by research assistants at these three times. Fasting blood samples were drawn at entry (gestational weeks 16.02±4.47, mean ± SD) and during the 3^rd^ trimester (gestational weeks 30.28±3.43). The accepted window for drawing the sample was +/− 2 weeks. Should the subject not appear, our policy was to have staff undertake a home visit to obtain the samples. A standardized protocol for biological specimen collection is used for the Camden Study which includes the serum preparation used in the current study. Briefly, whole venous blood was collected into red topped silicone coated Vacutainer tubes (Becton Dickinson, Franklin Lakes, NJ), allowed to stay at room temperature for 30–60 minutes and spun at 1500× g for 10 minutes in a 4°C centrifuge. The supernatant (serum) was immediately transferred into clean polypropylene cryovials and stored at −70°C. The serum samples were aliquoted and not thawed until assayed. The protocol for samples collected during home visits was the same.

Ethnicity was self-defined. Blood pressure was measured by clinic staff at each visit and abstracted from the medical records. BMI (kg/m^2^) was computed based on self reported pregravid weight; height was measured at entry to prenatal care. Maternal obesity was defined as BMI ≥30.

### Definition of Cases and Controls

Preterm delivery is defined as delivery at <37 completed weeks of gestation based upon the last menstrual period confirmed or modified by ultrasound evaluation [8], [20]. Detailed information identifying women with spontaneous preterm delivery, and medically indicated preterm delivery was obtained from the prenatal, labor, deliver and newborn records. Information on reproductive history including prior preterm delivery as well as the medical events during the current pregnancy was also obtained by interview and/or abstracted from clinical records.

Preeclampsia is a common condition (<8%) associated with medically indicated preterm delivery and identified by systolic blood pressure ≥140 mmHg or diastolic blood pressure ≥90 mm Hg after 20 weeks' gestation in a previously normotensive woman accompanied by new-onset proteinuria (>300 mg/24-hour from a 24-hour urine collection or ≥1+ protein by dipstick in two samples at least 4-hour apart) [21].

The cases of preterm delivery included all women who delivered preterm (n = 240) from the underlying cohort. Spontaneous preterm delivery was defined by the presence of intact membranes, regular contractions and the absence of induction of labor or an elective caesarean section. Preterm premature rupture of membrane (PROM) was defined as rupture of membranes before the onset of labor. Women with spontaneous labor and PROM leading to preterm delivery were combined into the spontaneous preterm group (n = 192). The primary reasons for medically indicated preterm delivery (n = 48) were gestational hypertensive disorders (n = 23), intrauterine growth restriction (IUGR) (n = 4), chorionmnionitis (n = 4), fetal distress (n = 4) and others (n = 13). Normal controls (n = 439) were randomly selected among women who delivered a term infant by SAS PROC SURVEYSELECT to assure that distribution of maternal characteristics was the same in cases and controls.

### Analytic Procedures

Serum concentrations of endothelial dysfunction biomarkers including sICAM-1, sVCAM-1 and sE-selectin were measured by commercial enzyme-linked immunosorbent assay (ELISA) (R & D Systems, Minneapolis, Minn). All samples were analyzed in duplicate. The sensitivity for the assays was 0.096 ng/ml, 0.6 ng/ml and 0.009 ng/ml for sICAM-1, sVCAM-1 and sE-selectin respectively. The within and between assay coefficient of variation (%) were 4.3/8.6 for sICAM-1, 4.7/5.9 for sVCAM-1, 3.6/8.2 for sE-selectin.

### Statistical Analysis

Maternal characteristics between cases and controls were compared by Student's t tests (for continuous variables) and x^2^ tests (for categorical variables). Analysis of variance was used to assess the significance of linear trend and compare mean levels of each endothelial dysfunction biomarker between cases and controls after controlling for potential confounding variables. Multiple logistic regression was used to estimate the Odds Ratio (OR) and 95% confidence interval (CIs) for elevated levels of each biomarker with preterm delivery. We divided each biomarker into quartiles and used logistic regression to estimate the OR of preterm delivery in each quartile using the lowest quartile as the reference category. Multiple polytomous logistic regression was used to estimate the association of elevated sE-selectin with preterm delivery with and without preeclampsia. This is an extension of traditional logistic regression which models multiple levels of an outcome so that all adjusted odds ratios are estimated in the same model. We also analyzed data with pregnancy complications (gestational diabetes mellitus (GDM) and preeclampsia) that impact endothelial function included or excluded.

We used separate models to examine endothelial dysfunction biomarkers in preterm delivery at entry and in the 3^rd^ trimester. Models were controlled for potential confounding variables including maternal BMI, age, parity, cigarette smoking, and ethnicity. Potential confounders were defined as those which altered the adjusted odds ratio or regression coefficient by at least 10%, and assessed by comparing crude and adjusted coefficients. Prior history of preterm delivery, a known risk factor for preterm birth, was adjusted in analysis when appropriate. The statistical significant level was defined as p <0.05. All statistical analyses were performed using SAS v.9.1 (SAS Institute, Inc., Cary, NC).

## Results

Maternal characteristics including age, BMI, parity, cigarette smoking, ethnicity, gestational age and blood pressure at entry were not significantly different between cases and controls (table 1). As expected, cases of preterm delivery had significantly shorter gestations (p<0.0001), infants with lower birth weights (p<0.0001); more of the cases had a prior history of preterm delivery (p<0.001) and more were complicated by preeclampsia (p<0.0001) or had increased 3^rd^ trimester blood pressure (p<0.0001) compared to controls. No significant difference in gestational diabetes prevalence was observed between cases and controls (p = 0.245).

**Table 1:** Clinical characteristics of preterm delivery cases and controls*.

Variable	Cases	Controls	p-value
	n = 240	n = 439	
Entry to care			
Age (yr)	22.82±5.93	22.10±5.20	0.23
BMI (kg/m^2^)	26.25±6.42	25.59±5.72	0.43
Obesity (BMI ≥30) n (%)	57 (23.75)	81 (18.45)	0.11
Nulliparas n (%)	86 (35.83)	175 (39.86)	0.30
Cigarette smoking n (%)	55 (22.92)	83 (18.91)	0.21
Ethnicity n (%)			
Hispanic	105 (43.75)	212 (48.29)	
African American	100 (41.67)	149 (33.94)	
Caucasian and other	35 (14.58)	78 (17.77)	0.13
Medicaid n (%)	238 (99.16)	427 (97.27)	0.24
Gestational age at entry (weeks)	14.10±4.80	14.21±4.91	0.79
Prior history of preterm delivery n (%)†	37 (24.03)	27 (10.23)	0.0002
Blood pressure			
Systolic blood pressure	112.90±13.60	111.80±11.5	0.20
Diastolic blood pressure	70.12±9.70	69.98±8.70	0.93
3^rd^ trimester and birth outcome			
Blood pressure			
Systolic blood pressure	119.83±15,41	115.50±10.93	<0.001
Diastolic blood pressure	76.43±12.90	72.20±9.02	<0.001
Singleton n (%)	228 (95.00)	438 (99.77)	<0.001
Gestational diabetes mellitus n (%)	14 (5.83)	38 (8.65)	0.249
Preeclampsia n (%)	47 (19.58)	39 (8.88)	<0.0001
Gestational age at delivery (weeks)	33.14±3.91	39.24±1.21	<0.0001
Infant birth weight (g)	2178±825	3330±435	<0.0001
			

*Data are mean ± SD or n (%). p-values are from ANOVA or chi-square test.

† Prior history of preterm delivery in parous women.

The correlations between concentrations at entry and the 3^rd^ trimester were 0.68, 0.60, 0.75 for sICAM-1, sVCAM-1 and sE-selectin respectively (p<0.0001 for each). Mean levels of fasting serum sICAM-1 and sVCAM-1 were significantly higher in all cases of preterm delivery and for cases with a spontaneous preterm delivery (n = 192) at entry (p<0.05 – p<0.001 for each) and in the 3^rd^ trimester (p<0.05 – p<0.001 for each). Concentrations of serum sE-selectin were not different between cases and controls in either early or later gestation (p>0.05 for each) (table 2). Although the concentrations of all three biomarkers were slightly increased during the 3^rd^ trimester compared to entry, none of the difference reached statistical significance in the cases or in the controls (p>0.05 for each). We also observed that the biomarker concentrations were increased in medically indicated preterm delivery compared to controls (p<0.05 for each). Differences did not reach statistical significance compared to spontaneous preterm cases except for sICAM-1 and sE-selectin in the 3^rd^ trimester (p<0.04 for each, table 2).

**Table 2:** Serum concentrations of endothelial dysfunction biomarkers in preterm delivery cases and controls (ng/ml)*.

	Cases			Controls	p-value †	p-value ‡	p-value§
	All Preterm (n = 240)	Spontaneous preterm (n = 192)	Medically Indicated preterm (n = 48)	(n = 439)			
**Entry to care**							
sICAM-1	217.05±4.63	215.50±5.15	227.33±10.70	203.29±3.40	0.018	0.037	0.026
sVCAM-1	550.96±8.34	551.94±9.06	551.40±19.60	512.99±6.13	0.0003	0.0006	0.056
sE-selectin	41.48±0.94	40.68±1.05	44.87±2.13	40.22±0.69	0.27	0.62	0.047
**3^rd^ trimester**							
sICAM-1	227.88±5.81	222.20±6.52	249.85±12.56∥	206.54±3.76	0.0025	0.043	0.001
sVCAM-1	567.22±10.63	558.93±11.66	595.83±23.32	522.68±6.84	0.0005	0.0054	0.003
sE-selectin	43.14±1.09	41.93±1.23	47.28±2.40∥	41.85±0.71	0.33	0.82	0.033

Abbreviations: sICAM-1, soluble intercellular adhesion molecule-1; sVCAM-1, soluble vascular cell adhesion molecule-1; sE-selectin, soluble E-selectin; same as in tables 3–6.

*Data are mean ± SE and adjusted for age, BMI, parity, ethnicity and cigarette smoking.

† All preterm delivery cases vs. controls.

‡ Spontaneous preterm delivery cases vs. controls.

§ Medically indicated preterm delivery cases vs. controls.

∥ Medically indicated preterm delivery cases vs. spontaneous preterm delivery cases p<0.05.

Elevated levels (highest quartile) of sICAM-1 and sVCAM-1 were positively associated with preterm delivery (p for trend <0.05 – <0.001 for each). At entry, the adjusted odds ratio (AOR) was 1.73 (95% CI 1.09–2.74) (table 3, model 1) for the highest quartile of sICAM-1 relative to the lowest quartile; and for sVCAM-1 the AOR was 2.17 (95% CI 1.36–3.46). When analyses were limited to cases with a spontaneous preterm delivery, these results again remained unchanged (table 3, model 2). We obtained similar data for the 3^rd^ trimester (table 4). However, sE-selectin was not related to preterm delivery in either early or later gestation (tables 3 and 4).

**Table 3:** Association of elevated endothelial dysfunction biomarkers levels with preterm delivery – Entry to care.

Biomarker	Quartile 1	Quartile 2	Quartile 3	Quartile 4	p for trend
n	169	171	169	170	
**sICAM-1**					
Median (IQR), ng/ml	113.4 (31.6–161.1)	189.5 (161.4–208.1)	229.4 (208.3–252.6)	288.7 (252.7–629.3)	
Model 1*					
OR (95% CI)	1.00	0.98 (0.63–1.55)	1.11 (0.71–1.75)	1.65 (1.06–2.58)	0.022
AOR (95% CI) *	1.00	1.13 (0.70–1.81)	1.23 (0.77–1.95)	1.73 (1.09–2.74)	0.020
Model 2 †					
OR (95% CI)	1.00	0.90 (0.55–1.48)	1.04 (0.64–1.69)	1.63 (1.02–2.62)	0.033
AOR (95% CI)	1.00	0.97 (0.58–1.62)	1.07 (0.65–1.76)	1.70 (1.05–2.76)	0.043
**sVCAM-1**					
Median (IQR), ng/ml	402.7 (199.4–443.4)	473.6 (445.3–504.7)	544.1 (505.7–585.0)	661.5 (586.4–1220.6)	
Model 1*					
OR (95% CI)	1.00	1.03 (0.65–1.64)	1.44 (0.91–2.26)	1.85 (1.18–2.90)	0.002
AOR (95% CI)	1.00	1.07 (0.67–1.72)	1.67 (1.05–2.66)	2.17 (1.36–3.46)	0.0003
Model 2 †					
OR (95% CI)	1.00	0.98 (0.59–1.64)	1.53 (0.94–2.49)	1.96 (1.21–3.18)	0.002
AOR (95% CI)	1.00	0.99 (0.59–1.68)	1.74 (1.05–2.88)	2.24 (1.35–3.70)	0.0003
**sE-selectin**					
Median(IQR), ng/ml	23.7 (1.7–29.2)	35.8 (29.2–40.2)	44.9 (40.2–49.4)	58.5 (49.5–92.5)	
Model 1*					
OR (95% CI)	1.00	1.00 (0.64–1.56)	0.76 (0.48–1.20)	1.27 (0.82–1.97)	0.52
AOR (95% CI)	1.00	0.99 (0.63–1.56)	0.75 (0.47–1.20)	1.22 (0.78–1.92)	0.64
Model 2 †					
OR (95% CI)	1.00	0.82 (0.51–1.32)	0.66 (0.41–1.07)	1.02 (0.64–1.63)	0.53
AOR (95% CI)	1.00	0.83 (0.51–1.34)	0.67 (0.41–1.09)	1.00 (0.62–1.61)	0.66

Abbreviations: IQR, interquartile range; OR, unadjusted odds ratio; AOR, adjusted odds ratio; 95% CI, 95% confidence interval.

*Model 1: All preterm cases and controls and was adjusted for age, BMI, ethnicity, parity and cigarette smoking.

† Model 2: Spontaneous preterm cases and controls with same adjustment.

**Table 4:** Association of elevated endothelial dysfunction biomarkers levels with preterm delivery: 3^rd^ trimester.

Biomarker	Quartile 1	Quartile 2	Quartile 3	Quartile 4	p for trend
n	160	160	160	161	
**sICAM-1**					
Median (IQR), ng/ml	120.2 (21.4–168.7)	192.7 (169.1–210.4)	228.0 (210.9–251.7)	282.2 (252.0–953.7)	
Model 1*					
OR (95% CI)	1.00	0.85 (0.51–1.41)	1.13 (0.69–1.85)	1.95 (1.22–3.13)	0.002
AOR (95% CI) *	1.00	0.94 (0.56–1.59)	1.27 (0.76–2.11)	2.17 (1.32–3.55)	0.001
Model 2 †					
OR (95% CI)	1.00	0.85 (0.49–1.47)	0.92 (0.53–1.59)	1.81 (1.09–3.01)	0.019
AOR (95% CI)	1.00	0.90 (0.51–1.57)	0.95 (0.54–1.67)	1.84 (1.08–3.13)	0.022
**sVCAM-1**					
Median (IQR), ng/ml	401.3 (134.3–438.3)	470.4 (439.0–512.6)	547.4 (512.8–595.7)	674.0 (597.0–1136.4)	
Model 1*					
OR (95% CI)	1.00	1.06 (0.64–1.78)	1.41 (0.86–2.33)	2.32 (1.43–3.76)	0.003
AOR (95% CI)	1.00	1.07 (0.63–1.81)	1.52 (0.92–2.54)	2.55 (1.55–4.19)	<0.0001
Model 2 †					
OR (95% CI)	1.00	1.07 (0.62–1.86)	1.23 (0.71–2.13)	2.01 (1.19–3.40)	0.008
AOR (95% CI)	1.00	1.06 (0.61–1.87)	1.29 (0.74–2.25)	2.15 (1.25–3.68)	0.004
**sE-selectin**					
Median(IQR), ng/ml	24.7 (5.3–31.1)	36.6 (31.2–41.1)	46.0 (41.2–52.2)	60.9 (52.3–95.3)	
Model 1*					
OR (95% CI)	1.00	1.10 (0.67–1.79)	0.90 (0.55–1.48)	1.50 (0.94–2.42)	0.166
AOR (95% CI)	1.00	1.15 (0.70–1.88)	0.89 (0.54–1.47)	1.52 (0.94–2.46)	0.18
Model 2 †					
OR (95% CI)	1.00	0.92 (0.55–1.56)	0.77 (0.45–1.32)	1.11 (0.66–1.86)	0.88
AOR (95% CI)	1.00	0.96 (0.57–1.63)	0.77 (0.45–1.32)	1.15 (0.68–1.95)	0.82

Abbreviations: IQR, interquartile range; OR, unadjusted odds ratio; AOR, adjusted odds ratio; 95% CI, 95% confidence interval. Thirty eight patient's samples or covariates were not available at the 3^rd^ trimester including 33 cases and 5 controls.

*Model 1: All preterm cases and controls and was adjusted for age, BMI, ethnicity, parity and cigarette smoking.

† Model 2: Spontaneous preterm cases and controls with same adjustment.

We performed additional analyses to confirm that the results were not driven by preeclampsia and/or GDM (table 5). Information on a prior history of preterm delivery was added to the covariates used in tables 3 and 4. Results were consistent when preeclampsia and GDM were controlled (table 5, model 1) or excluded (table 5, model 2) at entry and the 3^rd^ trimester.

**Table 5:** Elevated sICAM-1 and sVCAM-1 levels and preterm delivery: after inclusion and exclusion of preeclampsia, gestational diabetes and twins

		Entry AOR (95% CI)		3^rd^ trimesterAOR (95% CI)	
		sICAM-1*	sVCAM-1*	sICAM-1*	sVCAM-1*
All preterm cases vs. controls	Model 1 †	1.71 (1.05–2.80)	1.64 (1.00–2.71)	2.10 (1.25–3.50)	1.93 (1.16–3.24)
	Model 2 ‡	1.70 (1.07–2.72)	2.12 (1.33–3.40)	2.12 (1.29–3.49)	2.44 (1.48–4.03)
Spontaneous preterm cases vs. controls §	Model 1 †	1.66 (1.00–2.77)	1.75 (1.03–2.97)	1.85 (1.07–3.20)	1.76 (1.01–3.08)
	Model 2 ‡	1.66 (1.02–2.71)	2.24 (1.35–3.70)	1.81 (1.06–3.10)	2.01 (1.17–3.47)

Abbreviations: AOR, adjusted odds ratio; 95% CI, 95% confidence interval.

*The AOR (95% CI) compares the highest quartile to the lowest quartile of each biomarker.

† Model 1: Models were adjusted for age, BMI, parity, cigarette smoking, ethnicity, prior history of preterm delivery, preeclampsia and gestational diabetes. Twins were excluded.

‡ Model 2. Models were adjusted for age, BMI, parity, cigarette smoking, ethnicity and prior history of preterm delivery. Patients with preeclampsia, gestational diabetes as well as twins were excluded.

§ Medically indicated cases of preterm delivery were excluded.

We separated preterm deliveries into those with and those without preeclampsia to determine associations with sE-selectin (table 6). Elevated sE-selectin concentration (the highest quartile vs. other quartiles pooled) was significantly associated with preterm delivery complicated with preeclampsia (at entry, AOR 2.32, 95% CI 1.22–4.40). Similar results were obtained at the 3^rd^ trimester where the highest quartile of sE-selectin was associated with more than a 3-fold increased risk of preterm delivery with preeclampsia (AOR 3.37, 95% CI 1.78–6.39). A similar association was noted among controls where there was a 2-fold increased risk of preeclampsia in the 3^rd^ trimester with high sE-selectin (AOR = 2.05, 95% CI 1.03–4.10) but not at entry (AOR = 1.49, 95% CI 0.70–3.21). Using the same stratification and comparisons, elevated sICAM-1 and sVCAM-1 levels were consistently and significantly associated with preterm delivery without preeclampsia (table 6). The exception was sVCAM-1. During the 3^rd^ trimester the highest quartile was associated with preterm delivery with (AOR 3.34, 95% CI 1.75–6.39) and without preeclampsia (AOR = 1.73, 95% CI 1.14–2.63).

**Table 6:** Elevated endothelial dysfunction biomarker concentrations and preterm delivery with and without preeclampsia*.

	Entry to Care		3^rd^ trimester	
	Highest quartile of biomarker †n (%)	AOR (95% CI) §	Highest quartile of biomarker ‡n (%)	AOR (95% CI) ∥
**sE-selectin**				
Preterm with preeclampsia (n = 47)	19 (41.3)	2.32 (1.22–4.40)	22 (50.01)	3.37 (1.78–6.39)
Preterm without preeclampsia (n = 193)	50 (25.91)	1.20 (0.81–1.78)	36 (24.66)	1.16 (0.75–1.81)
Controls (n = 439)	99 (22.15)	1.00	100 (22.77)	1.00
**sICAM-1**				
Preterm with preeclampsia (n = 47)	10 (21.28)	1.06 (0.50–2.26)	9 (20.45)	1.10 (0.50–2.44)
Preterm without preeclampsia (n = 193)	63 (32.64)	1.66 (1.12–2.44)	57 (38.78)	2.38 (1.57–3.62)
Controls (n = 439)	94 (21.41)	1.00	95 (21.64)	1.00
**sVCAM-1**				
Preterm with preeclampsia (n = 47)	13 (27.66)	1.50 (0.75–2.99)	20 (46.51)	3.34 (1.75–6.39)
Preterm without preeclampsia (n = 193)	61 (31.66)	1.67 (1.14–2.45)	46 (31.29)	1.73 (1.14–2.63)
Controls (n = 439)	95 (21.64)	1.00	94 (21.40)	1.00

Abbreviations: AOR, adjusted odds ratio; 95% CI, 95% confidence interval.

*Models were adjusted for age, BMI, parity, ethnicity and cigarette smoking.

† Highest quartile of sE-selectin (≥49.43 ng/ml), sICAM-1 (≥252.72 ng/ml) and sVCAM-1 (≥585.04 ng/ml) at entry vs. other quartiles pooled for each biomarker.

‡ Highest quartile of sE-selectin (≥52.21 ng/ml), sICAM-1 (≥252.30 ng/ml) and sVCAM-1 (≥596.29 ng/ml) at 3^rd^ trimester vs. other quartiles pooled for each biomarker.

§ p for trend <0.05.

∥ p for trend <0.01.

## Discussion

Our purpose was to determine if endothelial dysfunction biomarkers which are known to be linked to CVD risk were also detectable in pregnant women who delivered preterm. This circumstance is analogous to the later risk of type 2 diabetes experienced by women with gestational diabetes. In our prospective nested case-control study of generally healthy pregnant women from Camden, we made three novel and important observations. Firstly, elevated serum levels of the endothelial dysfunction biomarkers sICAM-1 and sVCAM-1 were positively associated with preterm delivery and spontaneous preterm delivery. These associations were independent of confounding by the usual risk factors such as cigarette smoking, preeclampsia and prior history of preterm delivery. Secondly, the associations were detectable as early as 16 week gestation (at entry to care) and the effects persisted to the 3^rd^ trimester. Finally, our data showed that elevated sE-selectin was not associated with all cases of preterm delivery but only when a preterm (cases) or a term (controls) delivery was complicated by preeclampsia. Our research thus suggests that the physiological demands of pregnancy act as a ‘stress test’. Women who fail the test may be predisposed to pregnancy complications (such as preeclampsia) or adverse pregnancy outcome (such as preterm delivery). However, failing the ‘stress test’ may also allow early identification of women with risk factors for chronic disease of later life. Our data also support the plausibility that endothelial dysfunction, a precursor to CVD, may be associated with pathogenic factors related to preterm delivery and preeclampsia.

The healthy endothelium secretes and expresses a number of substances that help to maintain vascular wall structure and vascular homeostasis [13]. Endothelial dysfunction, a shift toward reduced vasodilatation, pro-inflammation, and increased thrombosis, has been observed in atherosclerosis [13], [14], [16]. While endothelial function can be directly measured with non-invasive methods e.g. blood flow-mediated vasodilation [6], [13], biomarkers of endothelial action (cellular, physiological or mediators) measured in peripheral blood are more feasible to obtain in large epidemiological studies. Endothelial cells can be activated, thus increasing the expression of cell surface adhesion molecules, by multiple factors including pro-inflammatory cytokines, hypercholesterolemia and oxidative stress [15], [17], [22], [23]. The key function of intercellular adhesion molecules (ICAM-1) is the regulation of leukocyte migration to the endothelium and leukocyte activation. The vascular cell adhesion molecule (VCAM-1) functions as a transmembrane receptor for the vascular endothelial cell membrane and recruits leukocyte to the sites of inflammation [13], [14]. Elevated levels of the soluble form of both markers are sign of endothelial activation, an early indication of atherosclerosis and type 2 diabetes mellitus [17]. Prior research supports the hypothesis that increased inflammation plays a causal role in the pathogenesis of preterm delivery and that inflammatory cytokines induce the endothelium to express adhesion molecules [13], [24], [25]. Previous studies linking endothelial dysfunction to risk of preterm delivery were limited, often by small sample size and yielded inconsistent results [26], [27]. A recent case-control study reported that blood endothelial dysfunction biomarkers at week 31 gestation were not different between women with threatened spontaneous preterm labor and controls [18].

Several large epidemiologic studies linking birth records to maternal death certificates showed that women with a history of preterm delivery were at increased risk of having CVD and cerebrovascular events like stroke as a cause of death [10]–[12]. Whether or not women who delivered preterm shared common risk factors with CVD was unclear. In this study, we found that elevated levels of sICAM-1 and sVCAM-1 in early and late gestation were consistently associated with preterm delivery after adjustment for potential confounding variables (tables 3 and 4). Similar results were obtained when cases of preeclampsia or of medically indicated preterm delivery were excluded (table 5). As far as we are aware, ours is the first study to document this relationship during pregnancy and suggests that factors that underlie preterm delivery may also be related to endothelial dysfunction in reproductive age women.

E-selectin is a member of the selectin family and its expression occurs in response to cytokines [13], [16]. E-selectin may act as a proinflammatory agent [28]. We did not find an association between high serum sE-selectin and risk of preterm delivery (tables 3 and 4). However, when we stratified cases of preterm delivery into those with and without preeclampsia, the highest quartile of sE-selectin at entry and during the 3^rd^ trimester was associated with preterm delivery complicated by preeclampsia (table 6). During the 3^rd^ trimester, a positive association was also observed among controls with preeclampsia. Thus, the underlying mechanism for preterm delivery complicated by preeclampsia could be a severe inflammatory response [15].

Previous studies noted impaired endothelial function in women with a history of preeclampsia compared to women with uncomplicated pregnancies, at a median interval of 3 years postpartum [15]. The changes included alterations in brachial artery diameter, and blood flow as well as increased blood levels of sE-selectin, with no difference in sICAM-1 [15]. There have been two additional reports of increased maternal plasma sE-selectin and/or sVCAM-1 [29], [30] in pregnant women where the biomarkers were measured after preeclampsia became overt. Our data extend these observations and link endothelial dysfunction before the diagnosis of preeclampsia to increased risk in women with preterm and term deliveries complicated by preeclampsia.

There are some limitations to this study. On one hand, our study population was young and most of the women were from ethnic minorities; consequently results may not be generalizable to other populations. On the other hand, African American women are at markedly increased risk for CVD as well as for preterm delivery and preeclampsia [31], [32]. These ethnic disparities are unexplained suggesting that unknown risk factors contribute. Thus our findings may have important implications for the prevention and treatment of CVD in high risk populations. In addition, factors such as dysregulation of glucose metabolism and oxidative stress, both of which impact the biomarkers of endothelial dysfunction, were not measured in the current study. Finally, it would be ideal if the cases were more homogeneous and limited to primiparous women, an issue which can be addressed in future research.

In summary, our research supports the hypothesis that endothelial dysfunction (as indicated by the increased concentration of three substances secreted by the endothelium), a common pathogenic precursor to CVD is detectable under the stress of pregnancy in women who deliver preterm (sICAM-1 and sVCAM-1) or have a preterm delivery complicated by preeclampsia (sE-selectin). Therapeutic interventions such as supplementation with ascorbic acid, coenzyme Q10 or statins suggest that endothelial dysfunction may be a modifiable risk factor [15], [33], [34]. Our findings underscore the importance of follow-up studies to determine if improving endothelial function may reduce later life CVD risk in women.
